# The incidence and mortality of ovarian cancer and their relationship with the Human Development Index in Asia

**DOI:** 10.3332/ecancer.2016.628

**Published:** 2016-03-24

**Authors:** Saeid Razi, Mahshid Ghoncheh, Abdollah Mohammadian-Hafshejani, Hojjat Aziznejhad, Mahdi Mohammadian, Hamid Salehiniya

**Affiliations:** 1 Reproductive Biotechnology Research Centre, Avicenna Research Institute, ACECR, Tehran, Iran; 2 Department of Epidemiology and Biostatistics, School of Public Health, Hamadan University of Medical Sciences, Hamadan, Iran; 3 Department of Social Medicine, School of Medicine, Rafsanjan University of Medical Sciences, Rafsanjan, Iran; 4 Malekan Health Centre, Tabriz University of Medical Sciences, Tabriz, Iran; 5 Department of Epidemiology and Biostatistics, School of Public Health, Isfahan University of Medical Sciences, Isfahan, Iran; 6 Minimally Invasive Surgery Research Centre, Iran University of Medical Sciences, Tehran, Iran; 7 Department of Epidemiology and Biostatistics, School of Public Health, Tehran University of Medical Sciences, Tehran, Iran; 8 Students’ Scientific Research Centre, Tehran University of Medical Sciences, Tehran, Iran

**Keywords:** ovarian cancer, Human Development Index, epidemiology, incidence, Asia

## Abstract

**Background:**

The incidence and mortality estimates of ovarian cancer based on human development are essential for planning by policy makers. This study is aimed at investigating the standardised incidence rates (SIR) and standardised mortality rates (SMR) of ovarian cancer and their relationship with the Human Development Index (HDI) in Asian countries.

**Methods:**

This study was an ecologic study in Asia for assessment of the correlation between SIR, age standardised rates (ASR), and HDI and their details, including life expectancy at birth, mean years of schooling, and gross national income (GNI) per capita. We used the correlation bivariate method for assessment of the correlation between ASR and HDI, and its details. Statistical significance was assumed if P < 0.05. All reported P-values were two-sided. Statistical analyses were performed using SPSS (Version 15.0, SPSS Inc.).

**Results:**

The highest SIR of ovarian cancer was observed in Singapore, Kazakhstan, and Brunei respectively. Indonesia, Brunei, and Afghanistan had the highest SMR. There was a positive correlation between the HDI and SIR (r = 0.143, p = 0.006). Correlation between SMR of ovarian cancer and HDI was not significant (r = 0.005, p = 052.0).

**Conclusion:**

According to the findings of this study, between the HDI and SIR, there was a positive correlation, but there was no correlation between the SMR and HDI.

## Introduction

Ovarian cancer is the eighth most common cancer among women, and it includes about 4% of all women’s cancers [1]. This disease has high morbidity and mortality rates among cancers of the reproductive system [1, 2]. According to global estimates 225,000 new cases were detected each year, and 140,000 people annually die from the disease [1]. Lifetime risk of ovarian cancer in women is one in 71, and the chance of dying from the disease is 1 in 95 [3].

Although the incidence and mortality of this disease is high, its aetiology is not fully understood [4]. Nowadays, a few factors associated with this cancer have been identified [5]. These are classified into three categories: protective factors (parity and use of contraceptive), risk factors (lack of birth, a history of family, and age), and factors such as lactation, age at menarche, and age at menopause, while causality between them and the ovarian cancer is still not proven [5–8].

The incidence and mortality of this disease varies in different regions of the world. This may be because of a difference in genetic and environmental factors. In recent years, cancer as the cause of death was well-known in high-income countries, but in the coming years, regardless of socioeconomic status, it will be a major cause of disease and death. One of the important factors is the Human Development Index (HDI) [9–11], which shows the socioeconomic status of people living in different countries [12].

Since the HDI shows the status of the various countries in terms of progress and development, and because of the increasing burden of non-communicable diseases, especially in low-income countries [1, 13–15], the aim of this study was to investigate the relationship between HDI and incidence of and mortality from ovarian cancer in Asian countries.

## Methods

This study was an ecologic study in Asia for assessment of the correlation between the age-specific incidence and mortality rate (ASR), and the HDI and its details, including life expectancy at birth, mean years of schooling, and gross national income (GNI) per capita. Data on the ASR for every Asian country for 2012 were obtained from the Global Cancer Project, which is available from (http://globocan.iarc.fr/Default.aspx), and data on the HDI was obtained from the Human Development Report 2013 [16], including specific information about the HDI and its details for every country in the word for 2012.

### The method of estimation of the age-specific incidence and mortality rates in the Global Cancer Project by the International Agency for Research on Cancer (IARC)

#### Age-specific incidence rate estimate

The methods of estimation are specific for each country, and the quality of the estimation depends upon the quality and the amount of information available for each country. In theory, there are as many methods as there are countries, and because of the variety and the complexity of these methods, an overall quality score for the incidence and mortality estimates combined is almost impossible to establish. However, an alphanumeric scoring system that independently describes the availability of incidence and mortality data has been established at the country level. The combined score is presented together with the estimates for each country with the aim of providing a wide indication of the robustness of the estimation. The methods to estimate the sex- and age-specific incidence rates of cancer for a specific country fall into one of the following extensive categories, in priority order:

1. Rates projected to 2012 (38 countries); 2. Most recent rates applied to 2012 population (20 countries); 3. Estimated from national mortality by modeling, using incidence and mortality ratios derived from recorded data in country-specific cancer registries (13 countries); 4. Estimated from national mortality by modeling using incidence mortality ratios derived from recorded data in local cancer registries in neighbouring countries (nine European countries); 5. Estimated from national mortality estimates using modeled survival (32 countries); 6. Estimated as the weighted average of the local rates (16 countries); 7. One cancer registry covering a part of a country as a representative of the country profile (11 countries); 8. Age/sex specific rates for ‘all cancers’ partitioned using data on the relative frequency of different cancers (by age and sex) (12 countries); and 9. The rates are those of neighbouring countries or registries in the same area (33 countries) [17, 18].

#### Age-specific mortality rate estimate

Depending on the degree of detail and accuracy of the national mortality data, six methods have been utilised in the following order of priority:

1. Rates projected to 2012 (69 countries); 2. Most recent rates applied to 2012 population (26 countries); 3. Estimations as the weighted average of regional rates (one country); 4. Estimations from national incidence by modeling, using country-specific survival (two countries); 5. Estimations from national incidence using modeled survival (83 countries); and 6. The rates of neighbouring countries or registries in the same area (three countries) [17–19].

#### Human development index

The HDI is a composite measure of indicators along with three dimensions: life expectancy, educational attainment, and command over the resources needed for a decent living. All groups and regions had notable improvement in all HDI components, with faster progress in low and medium HDI countries. On this basis, the world is becoming less unequal. Nevertheless, national averages hide large variations in human experience. Wide disparities remain within countries, both in the North and the South, and income inequality within and between many countries has been rising [16].

## Statistical analysis

In this study, we used the correlation bivariate method for assessment of the correlation between age ASR and HDI, and its details including life expectancy at birth, mean years of schooling, and gross national income (GNI) per capita. Statistical significance was assumed if P < 0.05. All reported P-values are two-sided. Statistical analyses were performed using SPSS (Version 15.0, SPSS Inc).

## Results

In general, Asian countries recorded 110,526 cases of ovarian cancer in 2012. Among these countries, the five countries with the highest number of cases were China (34,575 cases), India (26,834 cases), Indonesia (10,238 cases), Japan (8921 cases), and Pakistan (3703 cases). The five countries included 84,271 cases (24/76%) of patients in Asia.

**Figure 1.**
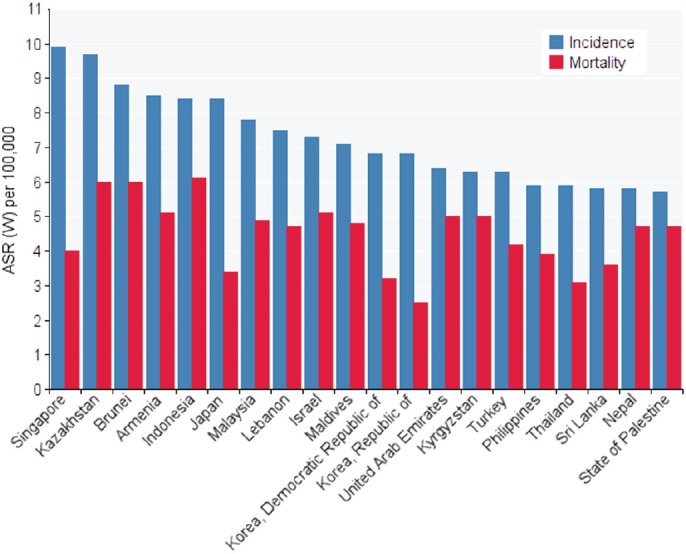
standardised incidence and mortality rates of ovarian cancer in Asia in 2012.

In Asian countries, five countries had the highest standardised incidence of ovarian cancer, as follows: Singapore with 9.9 per 100,000, Kazakhstan with 7.9 per 100,000, Beruni with 8.8 per 100,000, Armenia with 5.8 per 100,000, and Japan with 4.8 per 100,000, respectively. The five countries with the lowest standardised rates of ovarian cancer were Tajikistan with 2 per 100,000, Uzbekistan with 2.1 per 100,000, Azerbaijan with 2.1 per 100,000, Turkmenistan with 2.6 per 100,000, and Vietnam with 2.6 per 100,000 (Table and Figure 1).

The number of 65,668 deaths because of ovarian cancer occurred in Asian countries in 2012. The greatest number of deaths were in India (19,549), China (14,676), Indonesia (7075), Japan (4986), and Pakistan (2726). The total number of deaths in the five countries was 49,012 (63/74%).

In Asian countries, the five countries with the highest standardised death rate of ovarian cancer were as follows: Indonesia 1.6 per 100,000, Brunei with 6 per 100,000, Kazakhstan with 6 per 100,000, Armenia with 5.1 per 100,000, and Israel with 5.1 per 100,000. The five countries with the lowest standardised mortality rate of ovarian cancer included Uzbekistan with 7.1 per 100,000, China with 1.7 per 100,000, Azerbaijan with 1.7 per 100,000, Tajikistanwith 1.8 per 100,000, and Vietnam with 1.9 per 100,000 (Table and Figure 1).

Table 2 shows values of the HDI and its components for each of the Asian countries. The Asian countries in terms of HDI are classified as follows: the three countries in the very high category, four countries in the high category, thirty-five countries in the middle category, three countries in the low category, and one country in the unknown category.

### Standardised incidence rate and HDI

The standardised incidence rate for ovarian cancer had a positive correlation with the HDI, which was statistically significant (p = 0.006).

**Figure 2.**
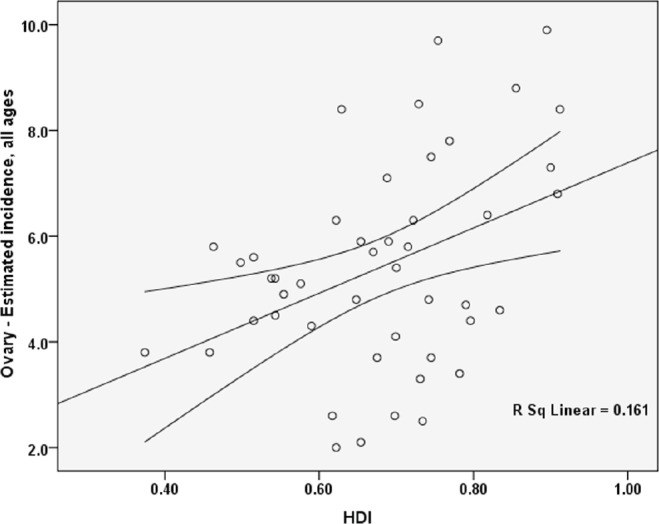
Correlation between HDI and standardised incidence rates of ovarian cancer in Asia in 2012.

There was a positive correlation between the components of the HDI and standardised incidence rate, so that a positive correlation was seen between the standardised incidence rate and life expectancy at birth (p = .006), the standardised incidence rate and the average years of schooling (0.143), and the standardised incidence rate and the level of income per person of population (p = 0.063) (Figure 2).

### The standardised mortality rate and HDI

**Figure 3.**
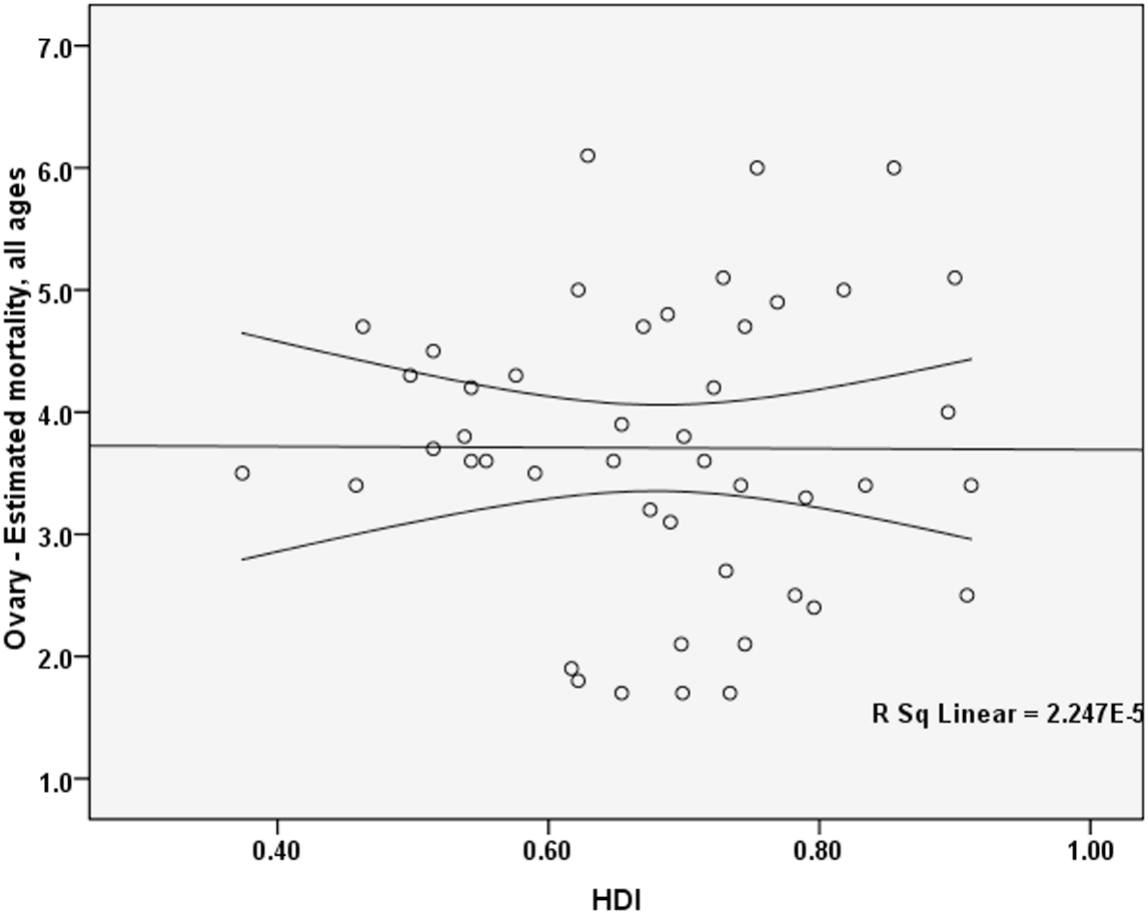
Correlation between HDI and standardised mortality rates for ovarian cancer in Asia in 2012.

The standardised mortality rate for ovarian cancer had a negative correlation with the Human Development Index and this association was not statistically significant (p = 0.975). Between the components of the HDI and standardised mortality rate, statistically significant correlation was not observed. So that a positive correlation between the standardised mortality and life expectancy at birth (p = 0.861), a negative correlation between the standardised mortality and mean years of schooling (0.373), and a positive correlation between the standardised mortality and the level of income for each person (p = 0.664) (Figure 3).

## Discussion

According to the findings of this study among Asian countries, Singapore, Kazakhstan, and Brunei had the highest standardised incidence rate of ovarian cancer. Studies also showed that Singapore has a high HDI, and the other two countries fell in the middle of the scale. Among these countries, the five countries with the highest rate of ovarian cancer included 84% of cancer cases related to Asian countries. Among these countries, Japan had a very high HDI and four other countries were in the middle of the scale. For standardised incidence rate, Singapore had the highest standardised incidence, and Tajikistan had the lowest. Our data in terms of the relationship between HDI and the standardised incidence rate showed that countries with HDI had a high-standardised incidence rate, and this finding was statistically significant.

The standardised mortality rate of ovarian cancer showed that five countries, including India, China, Indonesia, Japan, and Pakistan, had the highest rate of mortality. Overall, the countries included 63.74% of all deaths from ovarian cancer. There was no significant correlation between the HDI and standardised mortality rate.

In the absence of screening for ovarian cancer, diagnosis of this disease in the advanced stages leads to this cancer being considered as a fatal disease [19]. Most of the patients are diagnosed in stage 3 (71%) or stage 4 (31%). In other words, identifying this disease is hard, and the survival rate of the disease is low [20]. In addition, it has higher mortality among gynaecological cancers [20].

We also found that in the past, most of the world’s population was made up of children and teenagers, but with ageing (increased longevity) and a decrease in the birth rate, the declining population in the future will be remarkable. Because of increasing life expectancy and decreasing birth rate, the world has become ageing, and problems such as non-communicable diseases, especially cancer in the coming years are increasing. Most of the Asian countries are young, and the pattern of western lifestyle in these countries is progressive. Therefore, this problem will have a particular impact on developing countries.

According to the World Health Organisation in 2012, the increasing rate of older populations may lead to an increase in new cancer cases up to 19.3 million in 2025. Most new cases (56.8%) and mortality (64.9%) will occur in developing countries [17]. It is estimated that cancer causes 12.6% of total deaths. It is the second cause of death after heart disease. It is expected that cancer deaths will exceed those because of heart disease [21].

Although an increased incidence was seen in most countries, the difference between developed and developing countries was significant. Although developed countries have the highest incidence rate of cancer, the mortality rate is higher in developing countries. A lack of resources in developing countries leads to late diagnosis of the disease which causes high mortality from cancer in these countries. For example, the incidence of breast cancer in Western Europe was 90 new cases per 100,000 people, while the annual incidence in eastern Africa was 30 per 100,000, and mortality from the disease in these two regions nearly equals 15 per 100,000(21).

The incidence of cancer in the different geographic regions can partly be attributed to differences in risk factors related to lifestyle [22]. Several factors affect the occurrence of cancer, including tobacco, alcohol, work-related factors, pollution, water pollution, food and nutrition, obesity, physical activity, infectious agents, and UV radiation [23]. Related risk factors in developed countries include smoking, the pattern of nutritional, and reproductive behaviours, and in developing countries infectious agents, but disease patterns are changing [22]. One of the important factors in reducing the incidence and mortality of cancer can be HDI. The scale evaluates the long-term progress in three areas of human development. It is a composite index of three basic dimensions of human development, including life expectancy at birth, educational attainment (based on a combination of adult literacy rate and primary to tertiary education enrolment rates), and income (based on GDP per head, adjusted for purchasing-power parity in US$) (United Nations Development Programme: Human Development Index [HDI] [24].

Because of the lack of screening for ovarian cancer, its identification occurs at advanced stages, improving standard criteria in HDI, and awareness about lifestyle modification can partly prevent the incidence and mortality of this deadly disease.

## Limitations

This was an ecological study. Result of this type of study should be interpreted at the population level, and ecological fallacy will occur if results are inferred and concluded at the individual level. Some other factors such as the number of reproductive and contraceptive factors that suppress ovulation, including gravidity, breast feeding, and oral contraception, reduce the risk of ovarian cancer, and gynaecologic surgeries including hysterectomy and tubal ligation were protective. Some of the environmental factors and medical conditions that increased risk of disease included talc use, endometriosis, ovarian cysts, and hyperthyroidism. However, in this study we do not have access to enough information on these factors to consider their role in the incidence and mortality of ovarian cancer in Asian countries. Therefore, it is suggested that in addition to ecological studies, in each of these countries, studies in the form of case-control or cohort studies should be done to determine the role of factors related to the incidence and mortality from this disease on an individual level.

## Conclusions

Based on the findings of this study, ovarian cancer as a fatal disease in countries with low HDI had an increasing trend. The increasing number of elderly people in these countries, the changes in the way of life in these countries, as well as lack of facilities for screening in turn leading to delayed diagnosis, will all contribute towards these countries having to face rising mortality from this disease. Therefore, it seems that health policy makers should make serious decisions in this area to deal with the increase in the incidence and mortality of ovarian cancer. Asian countries are mostly developing countries, and the Western lifestyle in these countries causes a rapid increase in the burden of non-communicable diseases. Preventive programmes should be the top priority in these countries. In general, it can be concluded that by improving the socioeconomic situation, we find the incidence and mortality from infectious diseases and cancers related to infection are reduced, but cancers which are more associated with lifestyle factors are in rise.

## Figures and Tables

**Table 1.** Number, crude and standardised incidence rates, and mortality from ovarian cancer in Asian countries in 2012 (sorted by age-standardised rate from the highest to lowest).

Ovary - Estimated incidence, all ages				Ovary - Estimated mortality, all ages			
**POPULATION**	**Numbers**	**Crude Rate**	**ASR (W)**	**POPULATION**	**Numbers**	**Crude Rate**	**ASR (W)**
Singapore	371	14.2	9.9	Indonesia	7075	5.8	6.1
Kazakhstan	939	11.0	9.7	Brunei	9	4.4	6.0
Brunei	16	7.8	8.8	Kazakhstan	608	7.1	6.0
Armenia	193	11.6	8.5	Armenia	130	7.8	5.1
Japan	8921	13.7	8.4	Israel	296	7.6	5.1
Indonesia	10,238	8.3	8.4	United Arab Emirates	42	1.7	5.0
Malaysia	1098	7.6	7.8	Kyrgyzstan	113	4.1	5.0
Lebanon	183	8.3	7.5	Malaysia	645	4.5	4.9
Israel	380	9.8	7.3	Maldives	5	3.1	4.8
Maldives	9	5.6	7.1	State of Palestine	54	2.6	4.7
Korea, Democratic Republic of	1089	8.7	6.8	Nepal	528	3.4	4.7
Korea, Republic of	2349	9.6	6.8	Lebanon	116	5.3	4.7
United Arab Emirates	72	2.9	6.4	Pakistan	2726	3.1	4.5
Kyrgyzstan	158	5.7	6.3	Myanmar	1040	4.2	4.3
Turkey	2400	6.4	6.3	Timor-Leste	12	2.1	4.3
Thailand	2689	7.6	5.9	Turkey	1588	4.3	4.2
Philippines	2425	5.0	5.9	Lao PDR	94	2.9	4.2
Sri Lanka	736	6.8	5.8	Singapore	166	6.4	4.0
Nepal	702	4.5	5.8	Philippines	1442	3.0	3.9
State of Palestine	78	3.7	5.7	Jordan	71	2.3	3.8
Pakistan	3703	4.2	5.6	Bhutan	10	2.8	3.8
Myanmar	1396	5.6	5.5	Bangladesh	2166	2.9	3.7
Jordan	111	3.5	5.4	Sri Lanka	487	4.5	3.6
Lao PDR	129	4.0	5.2	India	19549	3.2	3.6
Bhutan	15	4.3	5.2	Syrian Arab Republic	262	2.5	3.6
Timor-Leste	16	2.7	5.1	Cambodia	219	3.0	3.6
India	26,834	4.4	4.9	Afghanistan	266	1.6	3.5
Syrian Arab Republic	385	3.7	4.8	Iraq	347	2.1	3.5
Iran, Islamic Republic of	1637	4.4	4.8	Japan	4986	7.7	3.4
Kuwait	31	2.7	4.7	Qatar	8	1.7	3.4
Qatar	14	3.0	4.6	Iran, Islamic Republic of	1076	2.9	3.4
Cambodia	300	4.1	4.5	Yemen	202	1.6	3.4
Bahrain	15	2.9	4.4	Kuwait	18	1.5	3.3
Bangladesh	2912	3.9	4.4	Korea, Democratic Republic of	551	4.4	3.2
Iraq	488	2.9	4.3	Mongolia	36	2.5	3.2
China	34575	5.3	4.1	Thailand	1431	4.0	3.1
Yemen	275	2.2	3.8	Oman	17	1.4	2.7
Afghanistan	346	2.1	3.8	Korea, Republic of	1054	4.3	2.5
Georgia	128	5.6	3.7	Saudi Arabia	190	1.5	2.5
Mongolia	46	3.2	3.7	Bahrain	9	1.8	2.4
Saudi Arabia	307	2.4	3.4	Georgia	82	3.6	2.1
Oman	25	2.1	3.3	Turkmenistan	47	1.8	2.1
Viet Nam	1254	2.8	2.6	Viet Nam	887	2.0	1.9
Turkmenistan	65	2.5	2.6	Tajikistan	43	1.2	1.8
Azerbaijan	141	3.0	2.5	Azerbaijan	93	2.0	1.7
Uzbekistan	275	1.9	2.1	China	14676	2.2	1.7
Tajikistan	57	1.6	2.0	Uzbekistan	196	1.4	1.7

**Table 2.** Human Development Index in Asian countries in 2012.

HDI	POPULATION	Human Development Index (HDI)	Life expectancy at birth	Mean Year of schooling	Gross national income (GNI) per capita
Very high	Japan	0.912	83.6	11.6	32545
	Korea, Republic of	0.909	80.7	11.6	28,231
	Israel	0.9	81.9	11.9	26,224
High	Singapore	0.895	81.2	10.1	52,613
	Brunei	0.855	78.1	8.6	45,690
	Qatar	0.834	78.5	7.3	87478
	United Arab Emirates	0.818	76.7	8.9	42,716
Medium	Bahrain	0.796	75.2	9.4	19,154
	Kuwait	0.79	74.7	6.1	52,793
	Saudi Arabia	0.782	74.1	7.8	22,616
	Malaysia	0.769	74.5	9.5	13,676
	Kazakhstan	0.754	67.4	10.4	10,451
	Georgia	0.745	73.9	12.1	5005
	Lebanon	0.745	72.8	7.9	12,364
	Iran, Islamic Republic of	0.742	73.2	7.8	10,695
	Azerbaijan	0.734	70.9	11.2	8153
	Oman	0.731	73.2	5.5	24,092
	Armenia	0.729	74.4	10.8	5540
	Turkey	0.722	74.2	6.5	13,710
	Sri Lanka	0.715	75.1	9.3	5170
	Jordan	0.7	73.5	8.6	5272
	China	0.699	73.7	7.5	7945
	Turkmenistan	0.698	65.2	9.9	7782
	Thailand	0.69	74.3	6.6	7722
	*Maldives*	0.688	77.1	5.8	7478
	Mongolia	0.675	68.8	8.3	4245
	State of Palestine	0.67	73	8	3359
	Philippines	0.654	69	8.9	3752
	Uzbekistan	0.654	68.6	10	3201
	*Syrian Arab Republic*	0.648	76	5.7	4674
	Indonesia	0.629	69.8	5.8	4154
	Kyrgyzstan	0.622	68	9.3	2009
	Tajikistan	0.622	67.8	9.8	2119
	Viet Nam	0.617	75.4	5.5	2970
	Iraq	0.59	69.6	5.6	3557
	*Timor-Leste*	0.576	62.9	4.4	5446
	India	0.554	65.8	4.4	3285
	*Cambodia*	0.543	63.6	5.8	2095
	*Lao PDR*	0.543	67.8	4.6	2435
	Bhutan	0.538	67.6	2.3	5246
	Bangladesh	0.515	69.2	4.8	1785
	Pakistan	0.515	65.7	4.9	2566
Low	*Myanmar*	0.498	65.7	3.9	1 817
	*Nepal*	0.463	69.1	3.2	1137
	Yemen	0.458	65.9	5.3	928
	*Afghanistan*	0.374	49.1	3.1	1000
Unknown	*Korea, Democratic Republic of*	–	–	–	–
